# Gamma Delta T-lymphocytes in Hepatitis C and Chronic Liver Disease

**DOI:** 10.3389/fimmu.2014.00400

**Published:** 2014-08-26

**Authors:** Neil Rajoriya, Joannah Ruth Fergusson, Joanna A. Leithead, Paul Klenerman

**Affiliations:** ^1^Peter Medawar Building for Pathogen Research, Oxford, UK; ^2^Liver Unit, Queen Elizabeth Hospital, Birmingham, UK; ^3^NIHR Biomedical Research Unit and Centre for Liver Research, University of Birmingham, Birmingham, UK; ^4^NIHR Biomedical Research Centre, Oxford Radcliffe Hospital, Oxford, UK

**Keywords:** gamma delta T-lymphocytes, γδ T-cells, CD161

## Abstract

Discovered 30 years ago, gamma delta (γδ) T-lymphocytes remain an intriguing and enigmatic T-cell subset. Although in humans they comprise a small fraction of the total circulating T-lymphocyte pool, they represent an important T-cell subset in tissues such as the liver, with roles bridging the innate and adaptive immune systems. The associations of γδ T-lymphocytes with chronic liver disease have been explored – however, there remain conflicting data as to whether these T-cells are pathogenic or protective. In patients with some forms of liver disease, their expansion in the periphery and especially in the liver may indeed help pathogen clearance, while in other conditions their presence may, in contrast, contribute to disease progression. γδ T-cells can also express CD161, a C-type lectin, and such cells have been found to be involved in the pathogenesis of inflammatory disease. CD161+ T-cells of diverse subsets are known to be enriched in the livers of patients with chronic hepatitis C. This article serves to provide a review of the γδ T-cell population and its role in hepatitis C and other chronic liver diseases, and also explores a potential role of the CD161+ γδ T-cells in liver diseases.

## Introduction: γδ T-Cells in Context

The liver is the largest organ in the body and also one of the most complex with multiple functional roles. Its dual vascular supply includes the portal vein and the hepatic artery, and thus, it has a prime location draining blood from the gut via the portal vein with a constant exposure to antigens (Ag) and bacterial products from the gut. However, it is not only harmful pathogens that challenge the liver, it must recognize and tolerate harmless foreign (food-derived) and self-Ags. Thus, the liver serves as a checkpoint to promote tolerance to self and non-pathogenic stimuli and protect against pathogens via the gut, and is therefore at risk of damage from viruses and bacteria.

The liver can be affected by many acute insults such as drugs, toxins, and acute viral infections; for example alcoholic hepatitis is one of the most florid manifestations of liver disease (1). When the insult is a continuous or recurrent over a period of time, a more chronic liver disease picture can develop leading initially to fibrosis and eventually, if the stimulus persists, cirrhosis. The most common causes of chronic liver disease globally include: hepatitis C virus (HCV), hepatitis B virus (HBV), alcoholic liver disease, non-alcoholic fatty liver disease (NAFLD)/non-alcoholic steatohepatitis (NASH), primary sclerosing cholangitis (PSC), and primary biliary cirrhosis (PBC). Chronic liver disease caused by any of these – with HCV a good example – can lead to a broad spectrum of liver injury from mild fibrosis to severe fibrosis, compensated cirrhosis and finally decompensated cirrhosis with major complications (e.g. esophageal variceal bleeding and spontaneous bacterial peritonitis) and hepatocellular carcinoma (HCC) development (2).

It is increasingly recognized that the liver has its own regional immune system providing a local immune-surveillance network, especially relevant to the gut. Blood from the gut drains via the superior and inferior mesenteric veins into the portal vein and then via the portal tracts and into the liver sinusoids and leaves via the central vein. In the liver sinusoids, there is a complex network of specialized immune-surveillance/antigen presenting cells (including liver sinusoidal endothelial cells, Kupffer cells, and hepatic stellate cells), which possess mechanisms allowing induction and recruitment of innate and adaptive responses. Irrespective of the causative agent of liver disease, once activated, the intrahepatic immune system is typically thought to play a dual role both in local host defense, and also in causing and/or propagating liver disease.

The protective role of intrahepatic cellular immune responses can be clearly seen in viral hepatitis. Spontaneous control of HCV infection is linked to the activation of intrahepatic cellular immunity and, conversely, the progression of HCV from an acute to chronic infection (occurring in the majority of cases) relies on evasion of cellular immunity (3). In acute HBV infection, a vigorous response with robust generation of interferon (IFN-γ) and tumor necrosis factor-alpha (TNF-α) can lead to clearance in 95% of cases in adults (4). Clearance of the virus in both these contexts is typically accompanied by a flare in the hepatocyte-derived amino transaminases, suggesting an aggressive immune mediated response within the liver. Indeed, the degree of liver damage correlates with the odds ratio of HCV clearance (5). The relevant intrahepatic lymphocyte (IHL) populations have been broadly characterized in humans, with the dominant intrahepatic population being the αβ T-cell populations. However, there also exist many other subsets and gamma delta (γδ) T-cells comprise 15% of mouse and human IHLs (6).

Gamma delta T-cells represent approximately 2–10% of all human T-cells in the peripheral blood (7) and are also found in diverse tissues, most abundantly in gut epithelial tissue, and also skin and bronchial epithelia. The position of γδ T-cells at epithelial barriers is strategic, i.e. continuously exposed to Ag and bacteria. A specific subset of CD161+ γδ T-cell has been implicated in inflammatory diseases such as multiple sclerosis (8, 9). CD161+ T-cells, however, have been found to be enriched in the livers of patients with chronic HCV (10–15) and thus the specific features of CD161+ γδ T-cells are of interest.

This article focuses on the γδ T-cell lymphocyte population in HCV, drawing also on data from other related chronic liver diseases.

## γδ T-Cells: The Basic Toolkit

Gamma delta T-cells were discovered after isolation of the TCR γ chain gene (16) and represent the subgroup of T-cells that have γ- and δ-glycoprotein chains linked by disulfide bonds rather than the “conventional” αβ chain. Large numbers of different receptors can be generated where different segments of genes: the variable (V), diversity (D), and joining (J) segments combine at random to produce numerous gene sequences – “VDJ recombination.” This re-arrangement allows the generation of different receptors to a variety of different Ags (17). γδ T-cells express CD3 – a complex forming part of the TCR, but are normally CD4/CD8 negative (double negative), although they may express variably CD4 or CD8 (18, 19). γδ T-cells, in contrast to αβ T-cells, do not require major histocompatibility complex (MHC) presentation of Ag. Two main subsets of γδ T-cells exist in humans determined by their Vδ chain re-arrangement (although there are six subsets in total in humans). Vδ1 cells are predominantly found in the gut and other peripheral tissues (such as lung, kidney, and spleen), whereas Vδ2 cells represent the major fraction of circulating γδ cells in blood (20).

## Vδ1 T-Cells

Gamma delta-T-cells in the gut epithelium (predominantly the Vδ1 subset) comprise up to 50% of the intra-epithelial lymphocyte population (21), a population that is expanded in certain disease states such as celiac disease (22). The Vδ1 T-cell population is also expanded in intracellular bacterial infections (*Mycobacterium tuberculosis* and *Listeria monocytogenes*), extracellular infections (*Borrelia burgdorferi*) and have been shown to proliferate in response to stimulation with Ags from cytomegalovirus (CMV) (23, 24). Vδ1-expressing γδ T-cells have also found to be expanded in the periphery in patients with acquired immunodeficiency syndrome (25). The Vδ1 subset recognize stress-inducible MHC class 1 chain-related Ags (MIC) A/B (26) or self-lipid presented Ags by CD1c (27). These γδ T-cells can also be modulated by toll-like receptor (TLR) ligands. TLR 1, 2, 3, 5, and 6 ligands can act in combination with TCR stimulation to enhance chemokine and cytokine production (28). The Vδ1 subset is not restricted to the gut, but found at other sites including the skin (29). It has recently been described that a subset of such cells can respond to CD1d-lipid complexes in a manner analogous to iNKT cells (30).

## Vδ2 T-Cells

The Vδ2 subset (also known as Vδ2Vγ9) are unique to humans and primates making up to 50–95% of the total γδ population in the blood and further expanding in acute infections such as *M. tuberculosis, Salmonella, Listeria, Brucella*, Malaria, and Toxoplasmosis even before other lymphocyte populations expand (31, 32). The Vδ2 subset can, however, also play an important role in local disease processes in barriers such as the skin (33) acting as a pro-inflammatory subset likely involved in the pathogenesis of psoriatic lesions in the skin, for example, reduced circulating levels of these cells are found in the periphery compared to healthy controls or patients with atopic dermatitis.

The Vδ2 subset recognizes small non-peptide pyrophosphomonoesters Ag, collectively known as “Phos-Ag” [the main one being the microbial compound (e)-4-hydroxy-3-methyl-but-2-enyl-pyrophosphate (HMB-PP) – an essential metabolite in pathogenic bacteria but not in the commensal human microbiota]. Bacteria synthesizing isopentenyl pyrophosphate (IPP) via the classical mevalonate pathway such as *Streptococcus* and *Staphylococcus* do not produce HMB-PP and thus do not recruit γδ T-cells, whereas other bacteria such as *L. monocytogenes* and the parasite *Toxoplasma gondii*, which synthesize IPP via the alternative non-mevalonate pathway do indeed produce HMB-PP and thus recruit γδ T-cells. The aminobisphosphonates (used in clinical practice for bone strengthening in osteoporosis by the inhibition of bone digestion by osteoclasts) are structurally related to IPP, and thus, act as Vδ2 agonists (34). There have been only a few single non-peptide ligands that have been shown to be both necessary and sufficient to stimulate γδ T-cells, and indeed T-cell activation by Phos-Ag often additionally requires cell-to-cell contact (7). Recent data have indicated that butyrophilin 3A1 is a surface molecule required for the presentation of Phos-Ag to γδ T-cells, although the exact nature of the presenting pathway still needs to be fully defined (35).

## γδ-T-Cells: Functions *In vivo*

In addition to conventional type 1 T-cell functions, recently γδ T-cells have been found to be a source of IL-17 (36) promoting granulopoiesis and neutrophil recruitment to aid pathogen clearance. The production of IL-17 by γδ cells in the gut epithelium can lead to tight junction formation and mucin secretion in the face of insults to the gut epithelium. They have also been found to produce IL-17 during TB infection in the lung (37). Along with IL-17, γδ T-cells may also produce IL-22, granzyme B, perforin, TNF-α, and IFN-γ (38–40). IL-17 producing γδ-T-cells may contribute to the efficacy of anticancer chemotherapy (41); chemotherapy leads to a rapid invasion of γδ-T-cells into the tumor bed, preceding accumulation of Tc-1 cytotoxic T-lymphocytes. The anticancer effect of the γδ-T-cells can be lost when there is a lack/deficiency of IL-17A or IL-1R1.

Cytotoxicity of γδ T-cells has been widely studied in the context of tumors. *Ex vivo* expanded γδ T-cells from healthy volunteers have been shown to be cytotoxic to high-grade glioblastomas *in vitro* and *in vivo* (42). γδ T-cells have also been shown to mediate killing of other tumor cells and represent an important effector of the immune system with an anti-tumor peripheral surveillance role (43). The Vδ2 T-cells are triggered by Phos-Ags (which are indeed increased in malignancy) and produce cytokines typical of Th-1, Th-2, or Th-17 cells (44–46), cross-talk with DCs (47) and also have a direct cytotoxic effect via: perforin/granzyme, Fas/FasL, TNF/TNF-R, and TRAIL-TRAIL-R pathways (29). The killing capacity of the Vδ2 T-cells was improved by pre-treatment of tumor target cells with aminobisphosphonates. The role of γδ T-cells in the future of anticancer (including HCC) therapy may be either via adoptive transfer (48) or *in vivo* stimulation and recruitment through the aminobisphosphonates (49).

## γδ T-Cells and Hepatitis

Gamma delta T-cells localize preferentially in the liver compared to blood (14) – thus, their contribution to liver disease remains of great interest (see Table 1). Kenna and colleagues (13) showed marked enrichment of γδ T-cells in normal liver specimens from healthy donors compared to blood. In their study, they found a clear enrichment of the Vδ3 subset (mean in liver 21%) compared to blood, where it is very rarely found (0.5%). In healthy donors, the dominant Vδ population was still found to be Vδ2, as in blood, but relatively enriched compared to Vδ1 cells.

**Table 1:** **Summary of γδ T-cell role and function in published studies in liver diseases**.

Reference	Area of hepatology	Site of interest	γδ T-cell population findings
Kenna et al. (13)	General	Intrahepatic vs. peripheral γδ T-cell population	Vδ3 T-cell population enriched compared to the periphery
Kanayama et al. (50)	General	Intrahepatic γδ T-cell population	Increased γδ T-cells in immunohistochemical staining of patients with chronic liver diseases
Kasper et al. (51)	Viral (HBV/HCV)	Intrahepatic γδ T-cell population	Equal frequencies of lobular infiltrates of αβ and γδ T-cells in patients
Tseng et al. (52)	Viral (HBV/HCV)	γδ T-cell generated from liver biopsy *in vitro*	Significant numbers of γδ T-cells in HCV/HBV infected patients compared to expanded cells from the non-virally infected liver
Agrati et al. (53)	Viral (HCV)	γδ T-cells from matched liver/blood samples	Specific compartmentalization of Vδ1 cells in preference to Vδ2 γδ expressed a memory/effector phenotype (CD62L− CD45RO+ CD95+) and produced IFN-γ and IL-4
Agrati et al. (82)	Viral (HCV)	Vδ2 T-cells on Huh7 hepatoma cells carrying the subgenomic HCV replicon	Activation of the Vδ2 cells was associated with a marked reduction of HCV RNA levels The inhibition of HCV was increased by the use of aminobisphosphonates
Pár et al. (55)	Viral (HCV)	Peripheral blood γδ T-cell population	Decreased percentage of Vδ2 cells, as well as reduced perforin-positive T-lymphocytes compared to controls or to those who had cleared HCV
Chen et al. (56)	Viral (HBV)	Peripheral blood γδ T-cell population	Peripheral Vδ2 T-cells negatively correlated with liver function tests IFN-γ and TCR-γδ T-cell cytotoxicity were lower in patients with chronic HBV compared to healthy controls
Malan-Borel et al. (57), Koshiba et al. (58)	Transplant	Peripheral blood γδ T-cell population	Increased numbers of γδ T-cells have been identified in the circulation of patients with stable liver graft function
Puig-Pey et al. (59)	Transplant	Peripheral blood γδ T-cell population	Increased total circulating γδ T-cell population in patients post-transplant vs. healthy controls (predominant Vδ1) T-cell population Physiological reversal of blood Vδ2 > Vδ1 was seen in patients with HCV Increased Vδ1 populations in the post-transplant setting as a consequence of certain chronic viral triggers Vδ1 cell population expressed CD4, CD8, NKG2D, perforin, and IL-17A
Martinez-Llordella et al. (60)	Transplant	Peripheral blood γδ T-cell population	Increased Vδ1 peripheral circulating cells in patients in whom immunosuppression could be withdrawn
			Genes encoding for γδ T-cells found in such patients when compared to those in whom immunosuppression could not be withdrawn
Ferri et al. (61)	Autoimmune liver disease	Peripheral blood γδ T-cell population	Comparable frequencies between the PBMCs of patients with AIH (irrespective of disease state-active vs. remission) and healthy control groups In AIH patients, reversal of the physiological Vδ2:Vδ1 ratio with more Vδ1 cells found in the periphery The number of Vδ1 cells producing IFN-γ was significantly higher in patients with AIH compared to healthy controls
Martins et al. (62)	Autoimmune liver disease	Peripheral blood γδ T-cell population	γδ T-cells elevated numbers in the periphery in PBC/PSC when compared to controls
Hoh et al. (63)	Liver cancer	Expanded *ex vivo* γδ T-cell population	γδ T-cells expanded *ex vivo* (with aminobisphosphonate) and able to recognize and lyse HCC (and hepatoblastoma) cell in a co-culture system
Bouet-Toussaint et al. (64)	Liver cancer	Expanded *ex vivo* γδ T-cell population	Vδ2 cells expanded *ex vivo* could lyse HCC cell cultures

The presence of γδ T-cells in chronic hepatitis biopsies has been explored by Kasper and colleagues (51). In biopsies from 18 HBV and 25 HCV patients, they found the predominant portal tract infiltrate to be αβ T-cells; however, the lobular infiltration frequencies between αβ and γδ T-cells were approximately equal. Tseng and colleagues (52) studied T-cell lines generated from HCV+ or HBV+ patient liver biopsies *in vitro* and found significant numbers of γδ T-cells compared to expanded cells from the non-virally infected liver. These γδ T-cells had high levels of non-MHC-restricted cytotoxicity activity against primary hepatocytes and also produced high levels of IL-8, IFN-γ, and TNF-α when activated by anti-CD3. Similar findings were described by Kanayama and colleagues (50), who found increased γδ T-cells in immunohistochemical staining of liver tissue from patients with chronic liver disease. Thus, although not the dominant T-cell infiltrate in the liver, the γδ T-cell population has been found to be enriched in the livers of patients with liver disease.

The intrahepatic γδ T-cell population was further described by Agrati and colleagues (53), who studied 35 matched liver/blood samples from patients with chronic HCV. There was a specific compartmentalization of Vδ1 cells in preference to Vδ2 within the liver with the cells expressing a memory/effector phenotype (CD62L− CD45RO+ CD95+). On mitogenic stimulation of these cells they produced IFN-γ and IL-4. A higher frequency of IFN-γ producing Vδ1 cells was associated with higher degree of necro-inflammation, suggesting that these cells may indeed contribute to intrahepatic pathogenesis and disease progression in HCV patients. Similar observations were made in HCV/HIV co-infected patients, correlating Vδ1 infiltration with hepatic inflammation even in the setting of HAART (54).

The same group (53) further analyzed the antiviral functions of the Vδ2 T-cells on Huh7 hepatoma cells carrying the subgenomic HCV replicon. Activation of the Vδ2 cells was associated with a marked reduction of HCV RNA levels. The neutralization of IFN-γ by antibodies revealed the importance of this cytokine in inhibiting HCV replication. The inhibition of HCV was increased by the use of aminobisphosphonates that activate γδ T-cells – thus, pointing toward a potential future immunotherapeutic strategy. These studies suggested that depending on the type of Vδ chain expressed by the γδ T-cells, the cells might either have a beneficial or deleterious effect in patients with chronic HCV infection.

Par and colleagues (55) studied γδ T-cell responses in blood in patients who had HCV disease compared to healthy controls, and those patients who had cleared the virus with treatment. Patients with chronic HCV showed a decreased percentage of Vδ2 cells, as well as reduced perforin-positive T-lymphocytes compared to controls or to those who had cleared HCV infection. These data again suggested that impaired function and number of the Vδ2 γδ T-cells (although measured here in the periphery) may be involved in viral persistence. The γδ T-cell populations have also been studied in patients with chronic HBV (56). In patients with chronic HBV, the percentage of Vδ2 T-cells negatively correlated with serum alanine aminotransferase, aspartate transferase, and bilirubin suggesting that reduction in the Vδ2 population in the periphery had a role in the chronicity of HBV infection. Also, IFN-γ and TCR-γδ T-cell cytotoxicity was lower in patients with chronic HBV compared to healthy controls.

Relevant data also come from the liver transplant setting; increased numbers of γδ T-cells have been identified in the circulation of patients with stable liver graft function (57, 58, 60). Similarly, other authors (59) identified an increase in the total circulating peripheral γδ T-cell population in patients post-transplant when compared to healthy controls. The main, stable, circulating fraction of γδ T-cells was the Vδ1 T-cell population irrespective of type of transplant (single liver or combined liver/kidney) or type of immunosuppression. Interestingly, the physiological reversal of the normal peripheral blood distribution (Vδ2 > Vδ1) was particularly seen in patients with HCV and CMV positivity (rather than Epstein–Barr virus or herpes simplex virus positive patients). Thus, it was proposed that patients may have increased Vδ1 populations in the transplant setting as a consequence of certain chronic viral triggers. This Vδ1 cell population expressed CD4, CD8, NKG2D, perforin, and IL-17A.

In another study, biological traits were compared between patients who had immunosuppressive tolerance (i.e., who did not develop rejection on immunosuppression withdrawal), those who required ongoing immunosuppression and also healthy controls (60). Genes encoding for γδ T-cells were found in those with immune tolerance and these individuals were found to have significantly greater numbers of circulating (Vδ1) γδ T-cells than either non-tolerant patients or healthy individuals. Thus, the γδ T-cells may indeed play an important role in the post-liver transplant setting, especially in patients who may manage to withdraw their immunosuppression. The Vδ1 subset may expand in the setting of post-transplantation due to chronic viral triggers in the face of immunosuppression, or potentially the relative loss of Vδ2 cells may reflect recruitment to the liver.

Gamma delta T-cells have also been studied in the context of autoimmune liver disease (61). The γδ T-cell population frequency was found to be comparable between the PBMCs of patients with AIH (irrespective of disease state, i.e., active vs. remission) and healthy control groups. However, interestingly they also observed a reversal in the physiological Vδ2:Vδ1 ratio with more Vδ1 cells found in the periphery (*p* = 0.001), as in post-liver transplantation population previously described (59). In patients with AIH, the γδ T-cell population expressed significantly more granzyme B and the number of Vδ1 cells (but not Vδ2) producing IFN-γ was significantly higher in patients with AIH compared to healthy controls.

Since their function has been established in responses to certain tumors, γδ T-cells have also been studied in targeting HCC – a feature of advanced liver disease associated with HCV (64). In a recent study (63), γδ T-cells expanded *ex vivo* with an aminobisphosphonate were able to recognize and lyse HCC cell lines in co-culture assays – thus, providing the rationale for targeted immunotherapy in patients with HCC in the future.

**Figure 1:**
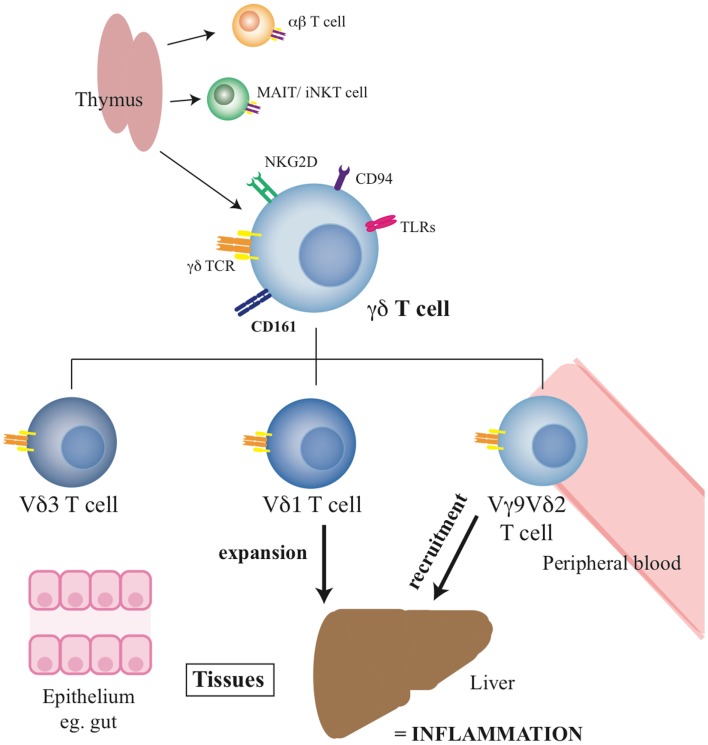
**Summary of γδ T-cell functions and the roles of specific subsets**. The diagram indicates the origin of the γδ T-cell pool, their phenotype, and the distribution of the key subsets in tissues.

Overall, these data suggest a clear disturbance of the normal distribution of γδ T-cells during chronic hepatitis C, with an apparently consistent redistribution of γδ T-cells to the liver, especially the lobular infiltrates. The impact of chronic hepatitis C appears to be differential according to the Vδ chain usage, although this needs to be confirmed by further studies. Such impacts are not restricted to HCV infection, and although a clear potential as a pro-inflammatory subset in chronic inflammation exists, in the absence of a relevant animal model where they can be depleted, this is not currently proven. The summary of the role and function of γδ T-cells in health and liver disease can be seen in Figure 1, with the published studies in the chronic liver disease setting summarised in Table 1.

## CD161 and γδ T-Cells

CD161 [also known as killer lectin receptor subfamily B member 1 (KLRB1)] is a C-type lectin membrane glycoprotein that is expressed on the majority of NK cells, and approximately 24% of peripheral T-cells (65). CD161 was originally identified as the human homolog of the NKRP1A glycoproteins expressed on rodent NK cells, demonstrating 46–47% homology with its rodent counterparts. It is composed of a disulfide-linked homodimer of 40 kDa subunits, and has been shown to be expressed on both αβ and γδ T-cells (66).

CD161 is present on CD4 and CD8 αβ T-cells with higher surface density on CD8 T-cells (65). T-cells expressing CD161 can be divided into further populations based on expression levels with high and intermediate levels (also known as CD161 “bright” and CD161 “mid” populations) (10, 67). CD161 T-cell expression has consistently been associated with a memory phenotype in adult circulations not only in αβ T-cell populations (67–70) but also in the γδ T-cell population (66). CD161 is expressed early in development: a subset of T-cells in fetal liver express CD161 as does a population of CD3+ thymocytes (65, 71). CD161+ CD4 T-cells and CD8+ T-cells have been found in umbilical cord blood (69, 71, 72).

CD161 expression is highly associated with liver-homing T cell populations (11, 14). CD161 positivity has been found particularly associated with mucosal-associated invariant T-cells (MAIT) (73) that are abundant in humans (comprising up to 45% of liver lymphocytes). Staining with anti-Vα7.2 mAb and either CD161 or IL-18R unequivocally identifies MAIT cells in the γδ−CD4−CD3+ compartment. CD161 is also expressed on a significant subset of HCV and HBV-specific T-cells (15), especially those in the IHL compartment. CD161-expressing CD4+ T-cells are also enriched in the liver (12). This cell population, like its CD8 counterpart, is associated with RORγt expression and the potential for IL-17 secretion (10, 69). Indeed, expression of CD161 marks all human T-cells, whether CD4+ or CD8+, αβ, or γδ, capable of producing IL-17 (66).

To date, there are no published studies of CD161+ γδ T-cells in liver disease. However, some intriguing data are emerging from other studies of tissue inflammation. CD161+γδ T-cells were first studied by Battistini et al. (74), with CD94 the most common NK receptor found on γδ T-cells. However, some γδ T-cells were found also to express CD161 and hence, it was proposed that the CD161 phenotype could be acquired *in vitro*; this was confirmed in culture experiments. On cross-linking CD161 on γδ T-cells with antibody, the γδ T-cells expressed large amounts of IFN-γ indicating the molecule could deliver a stimulatory signal.

These data reveal that γδ T-cells are capable of regulation via both the TCR and NK receptors, such as CD161, and hence act in both adaptive and innate manners, respectively. In this way, γδ T-cells have been described as part of the family of innate-like lymphocytes, which includes NKT and MAIT cells. Recently, MAIT cells have been described to respond in an innate-like manner to cytokine stimulation, independently of the TCR. The combination of IL-12 and IL-18 induced IFNγ from the CD161++ CD8+ T cell subset, including both MAIT and non-MAIT cells (75). Innate-like γδ T-cells, which similarly respond selectively to IL-12 and IL-18, have also been identified by Wencker et al. (76). This TCR-independent response may suggest a mechanism by which γδ T-cells can be activated in diverse infection settings, even in the absence of specific ligands. Indeed, response of γδ T-cells to various cytokines, and cytokine combinations, has previously been reported (77, 78).

In addition to a costimulatory role, prior experiments have also shown an involvement of CD161 in trans-endothelial migration (79), with CD161+ CD4 T-cells migrating further across endothelial cell monolayers compared to CD161−ve CD4 T-cells, an effect blocked by an anti-CD161 monoclonal antibody. CD161 expression was also found to be up-regulated on Vδ2 cells when cultured with IL-12 [known to up-regulate CD161 (80)]. Transmigration was then studied by culturing γδ T-cells with IL-2 or IL-12, and on day 6 assessing transmigration through HUVEC monolayers using a double transwell system. Vδ2 cell transmigration was higher and faster and enhanced by culture with IL-12. When blocking the cells with an anti-NKRP1A (191b8) monoclonal Ab, there was a significant reduction in trans-endothelial migration. It was postulated that the CD161+ γδ T-cells did indeed localize to sites of inflammation, and on exposure to IL-12 up-regulate expression of CD161. In contrast, further experiments revealed that the Vδ1 subset used platelet endothelial cell adhesion molecule (PCAM1 or CD31: an adhesion molecule involved in lymphocyte extravasion), rather than CD161, for trans-endothelial migration (81).

**Figure 2:**
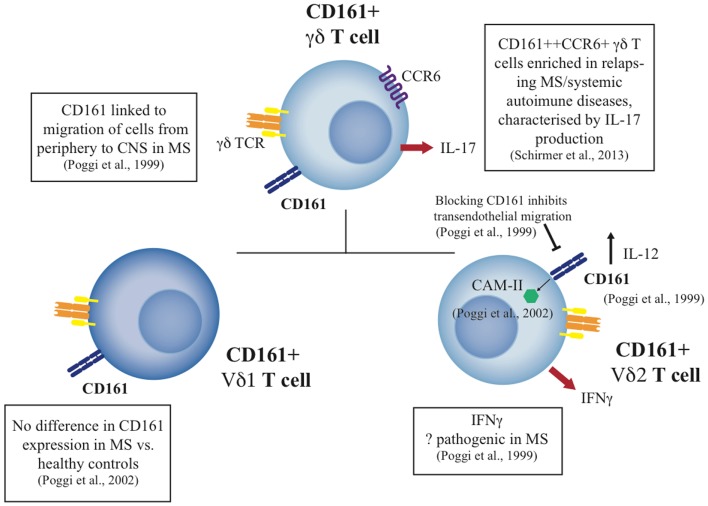
**Summary of CD161+ γδ T cell studies to date in health and disease**. The diagram indicates the phenotype and function of CD161+ γδ T cell subsets, including the Vδ1 and Vδ2 subsets, and their potential role in disease where published data exist.

These data indicate a specific functional role for CD161 on γδ T-cells, notably the Vδ2 cell subset, and a specific set of functions on CD161+ γδ T-cells in tissue inflammation. Since CD161 appears to be a general marker for liver-homing populations, the specific role of CD161+ γδ T-cells in liver disease is therefore of some interest for future studies. A summary of the role of CD161+ γδ T-cells can be seen in Figure 2.

## Conclusion

It is clear that although a small non-dominant T-cell subset in the human T-cell lineage, γδ T-cells play an important role in defense against foreign pathogens. Their role as innate sentinels, guarding against microbial invasion places them as a key T-cell subset in the battle against pathogens not only in the gut at an epithelial level but also, or potentially moreso, in the liver. Evidence is emerging that depending on the type of liver disease, γδ T-cells may play a role in disease pathogenesis. They have been shown to be effective killers of cancer cells and thus patients with HCV-driven HCC may be amenable to γδ T-cell-based immunotherapy.

Intriguingly, depending on their Vδ chain expression, the cells may provide protection or induce tissue damage in the liver; in particular, Vδ1 T-cells correlate with a higher necroinflammatory score in patients with chronic HCV (53). Further delineation of the relevant subsets may be of relevance in future. We postulate this particularly applies to CD161 expression as this has been linked to distinct functional attributes of the T cell, including pro-inflammatory IL-17 production, and is also closely associated with liver-homing populations. Finally, further studies of these and related intrahepatic T cell populations are relevant not only to chronic HCV but also to other chronic liver disease settings, especially those where therapeutic interventions are more limited.

## Conflict of Interest Statement

The authors declare that the research was conducted in the absence of any commercial or financial relationships that could be construed as a potential conflict of interest.
